# Evaluation of CareStart™ Malaria HRP2/pLDH (Pf/pan) Combo Test in a malaria low transmission region of Senegal

**DOI:** 10.1186/s12936-017-1980-z

**Published:** 2017-08-10

**Authors:** Mamadou Alpha Diallo, Khadim Diongue, Mouhamadou Ndiaye, Amy Gaye, Awa Deme, Aida S. Badiane, Daouda Ndiaye

**Affiliations:** Laboratoire de Parasitologie-Mycologie, Université Cheikh Anta Diop de Dakar, Avenue Cheikh Anta Diop, Fann, BP 5005, Dakar, Senegal

**Keywords:** CareStart™RDT, Microscopy, PCR, Sensitivity, Specificity, Diagnosis, Malaria

## Abstract

**Background:**

This study was initiated from the observation that prevalence of malaria obtained with rapid diagnostic test (RDT) (CareStart™Malaria HRP2/pLDH Combo Test) was higher than in microscopy in a malaria low transmission area of Senegal. PCR was then performed to evaluate the performance of the RDT compared to microscopy in clinical settings.

**Methods:**

The study included 215 patients suspected of malaria in two peri-urban area of Dakar. Finger-pick blood samples were tested using RDT (CareStart™Malaria HRP2/pLDH Combo Test). Venous blood samples were collected for light microscopy and PCR (gold standard). Sensitivity, specificity, positive predictive value (PPV), and negative predictive value (NPV) were calculated as performance characteristics.

**Results:**

Considering PCR as the gold standard, CareStart™RDT showed high sensitivity (97.3%) and specificity (94.1%) with PPV and NPV of 97.3 and 94.1%, respectively, while microscopy had a sensitivity and specificity of 93.2 and 100%, respectively, and PPV and NPV of 100 and 87.2%, respectively.

**Conclusions:**

Malaria CareStart™RDT test demonstrated a superior sensitivity compared to microscopy, which is the gold standard for malaria diagnosis. CareStart™RDT could be a useful tool in individuals suspected of malaria even in areas where prevalence is low.

## Background

Light microscopy detecting human malarial parasites in Giemsa-stained thick and thin blood films still remains the gold standard for malaria diagnosis [1]. Although with good sensitivity in diagnosing malaria, its reliability relies upon good-quality slide preparation and well-trained staff in parasite detection and differentiation, especially at low parasite densities [2]. In many peripheral health centres of endemic countries, microscopy is not available [3] due to limited resources or lack of expertise [4]. Simpler and rapid methods of identification of parasites may be useful for prompt diagnosis and appropriate treatment especially in peripheral health posts where microscopy is not available [5].

Malaria rapid diagnostic tests (RDTs), based on immunochromatographic parasite antigen detection, are of great value in endemic regions. Since 2010, WHO has recommended either RDT or microscopy confirmation of suspected malaria cases before treatment [3]. However, it remains unclear whether RDTs are useful in field conditions in low transmission areas [6] since their reliability is limited by the lack of detection of low-density parasitaemia [7]. Recently, an anecdotal observation indicated that there were more positive results with RDT (CareStart™Malaria HRP2/pLDH Combo Test) than with microscopy in a peri-urban region of Dakar where malaria transmission is low. CareStart™RDT Pf/Pan is one of the RDT being used in Senegal accordingly to the National Malaria Control Programme (NMCP).

The objective of this study was to compare CareStart™ RDT to microscopy performed by a WHO-certified level 1 microscopist and determine the test characteristics using PCR as gold standard.

## Methods

### Study design

The study was carried out in two sub-urban sites of Dakar (Pikine and Rufisque) from October to November 2016. Malaria transmission in Dakar is low, with parasitaemia prevalence estimate of 1.3% and a typical urban malaria pattern [8]. Pikine and Rufisque are characterized by unorganized urban growth. Malaria transmission in Dakar is heterogeneous in space and highly focal. Short rains occur from August to November. During this period breeding sites appear in tyres, step tracks, puddles, ditches, and garbage cans, or in debris on construction sites [9].

The study patients were enrolled if they presented with clinically suspected malaria on the basis of fever or history of fever in the previous 48 h to the health clinics in the study areas. Blood samples were collected for RDT and microscopy, and a filter paper collected for PCR. Inclusion criterion for this study was individuals with fever (suspicion of malaria) and without clinical sign of severe malaria.

### Microscopy evaluations

All parasitological examinations were carried out in Aristide Le Dantec Hospital Laboratory, the national reference laboratory of Senegalese National Malaria Control Programme. The laboratory hosts the external competency assessment (ECA) centre for African Francophone countries. Thick and thin blood smears were prepared from collected venous blood samples. Both thick and thin films were made in the same slide. The thin films were fixed in methanol. The slides were stained in 10% Giemsa solution for 15 min. Stained slides were read by two WHO-certified level 1 microscopists in the laboratory. The number of parasites was counted against 200 leucocytes when more than 100 parasites were counted, and 500 leucocytes were required when fewer than 100 parasites were counted. Quantification of parasite density was estimated by assuming 8000 leucocytes/μL of blood. The result was considered negative if no parasite was detected after examining 200 microscopic fields at 1000× magnification. The technician was blinded to the results of the RDT. In case of discordant results between the two readers, a third expert reader was used.

### Rapid diagnostic test (RDT)

The same patients were tested for malaria parasites using CareStart™Malaria HRP2/pLDH Combo Test in the fields (Lot Number: MR15A06). CareStart™RDT is an immunochromatographic test coated with monoclonal antibodies in two separate bands, one recognizing the specific histidine-rich protein-2 (HRP-2) associated with the presence of *Plasmodium falciparum* and the other detecting presence of pan malaria-specific antigen (pLDH) of all malarial parasites species. Five microlitre of blood was drawn using a loop provided with the device. The test preparation and interpretation was done following manufacturer’s instructions. Positive results indicate the presence of two or three bands (including control test line). For negative results, only the control line appears. Results were observed and number of visible line was recorded.

### PCR assay

From the collected venous blood, two drops of blood were spotted onto filter paper, individually stored in a plastic bag and sent to the Parasitology Laboratory of Le Dantec University Hospital for PET-PCR assay. DNA was extracted using the QIAamp blood kit (QIAGEN™) according to the manufacturer’s instructions and stored at 4 °C until processed. The *Plasmodium* PET-PCR reaction was performed as previously described [10, 11]. Samples with a CT value of 40.0 or below were considered positive.

### Data analysis

Data were processed in Excel (version 15.27) and analysed using STATA. Diagnostic performance was determined by calculating the test sensitivity (Se), specificity (Sp), predictive values (PPV and NPV), with 95% CI. PET-PCR was used as the reference method. McNemar Chi square analysis was used to determine the significance differences between Se, Sp, NPV and PPV. The p values less than 0.05 were considered statistical significance.

### Ethical consideration

Oral informed consent was obtained from the selected patients. Ethical approval with the reference number was obtained from Senegalese National Ethic Committee of Ministry of Health.

## Results

A total of 215 blood samples collected from malaria suspected patients were analysed. The sex ratio was 1.56:1. The mean age was 23 years (range 4–77 years). Among the 215 samples tested, the PPV was 137 (63.7%), 146 (67.9%) and 144 (68.4%) using microscopy, CareStart™RDT and PCR, respectively. Overall, CareStart™RDT detected more positive samples than microscopy and PCR. Species of malarial parasites identified in all study participants were *P. falciparum*. The parasite density ranged from 16 to 341,090 parasites/μL of blood with a mean of 36,823 parasites/μL by microscopy.

When compared to PCR, CareStart™RDT test showed better Se (97.3%) than microscopy (93.2%) (p = 0.0143) and better NPV, 94.1 versus 87.2% (p = 0.0251). However, better Sp (100%) and PPV (100%) were noted in microscopy versus RDT (94.1 and 97.3%, respectively) (p = 0.0455 and p = 0.0487, respectively) (see Table 1). PCR was used to resolve the discrepancies between microscopy and CareStart™RDT. There were 201 (93.5%) agreement results between all three methods. Thus, discrepancies were noted in 14 samples (6.5%). Microscopy was not able to detect 10 samples (4.7% of false negative microscopy). Among those 10 samples, CareStart™RDT also failed to detect four samples (false negative RDT) but correctly detected six samples. The four remaining samples were correctly read as negative by microscopy but were positive by RDT (false positive RDT). On false positive RDT, only HRP2 bands appeared. There was no false positive result with microscopy. In summary, CareStart™RDT showed less false negative but more false positive results compared to microscopy (see Table 2).

**Table 1:** Performance characteristics of microscopy and RDT compared to PCR

	PCR		Total
	Positive	Negative	
Microscopy			
Positive	137	0	137
Negative	10	68	78
Total	147	68	215
		IC95%	
Sensitivity (%)	93.2	[87.8–96.7]	
Specificity (%)	100.0	[95.7–100]	
PPV (%)	100.0	[97.8–100]	
NPV (%)	87.2	[77.7–93.7]	

	PCR		Total
	Positive	Negative	
CareStart™RDT			
Positive	143	4	147
Negative	4	64	68
Total	147	68	215
		IC95%	
Sensitivity (%)	97.3	[93.2–99.3]	
Specificity (%)	94.1	[85.6–98.4]	
PPV (%)	97.3	[93.2–99.3]	
NPV (%)	94.1	[85.6–98.4]	

	Test statistic	p values	
Sensitivity comparison	6.0	0.0143	
Specificity comparison	4.0	0.0455	
PPV comparison	3.9	0.0487	
NPV comparison	5.0	0.0251

**Table 2:** Discrepancies results between microscopy, RDT and PCR

Microscopy result	CareStart™RDT result	RDT bands	PCR result
Negative	Negative		Positive
Negative	Negative		Positive
Negative	Negative		Positive
Negative	Negative		Positive
Negative	Positive	HRP2 only	Negative
Negative	Positive	HRP2 only	Negative
Negative	Positive	HRP2 only	Negative
Negative	Positive	HRP2 only	Negative
Negative	Positive	HRP2 only	Positive
Negative	Positive	HRP2 only	Positive
Negative	Positive	HRP2 + LDH	Positive
Negative	Positive	HRP2 + LDH	Positive
Negative	Positive	HRP2 + LDH	Positive
Negative	Positive	HRP2 + LDH	Positive

Among the 147 RDT positive samples, only 47 presented LDH band (plus HRP2 band), giving an overall LDH positivity rate of 32.0%. This positivity rate was even less (17.4%) when parasite densities were less than 1000 (see Table 3).

**Table 3:** Analysis of RDT results according to the appearing bands

Parasite density	RDT bands		Total	LDH positivity rate (%)
	HRP2 + LDH	HRP2 only		
0	6	4	10	60
]0; 1000]	4	19	23	17.4
]1000; 10,000]	11	20	31	35.5
]10,000; 30,000]	10	30	40	25.0
]30,000; 50,000]	6	7	13	46.2
]50,000; +]	10	20	30	33.3
	47	100	147	32.0

## Discussion

RDTs have been developed especially for their ease of use in remote settings in endemic countries [1, 12]. However, many drawbacks have been reported with RDTs, especially relating to their sensitivity. In the literature, limit of detection of RDTs is estimated to be around 100 parasites/μL [4].

Several RDTs are commercially available with various quality of performance [12–14]. Thus, WHO requires all RDTs to reliably detect at least 100 parasites/μL and to present a minimum sensitivity of 95% and minimum specificity of 90% compared to traditional microscopy. Many studies have reported low sensitivity of RDTs when parasitaemia becomes lower than 200 parasites/μL [15]. Such low parasitaemia usually occur in malaria low transmission areas.

In a clinical health centre of sub-urban of Dakar (where low transmission of malaria occurs), it was reported that CareStart™RDT gave more positive results than microscopy performed by average microscopists. The aim of this study was to compare CareStart™RDT to microscopy using PCR as gold standard. The results of the study confirmed the observation noted by health-centre microscopists. In fact, CareStart™RDT showed better sensitivity than microscopy although specificity was better with the latter.

The CareStart™Malaria HRP2/pLDH (Pf/pan) Combo Test is a three-band RDT that detects HRP2 and pan-pLDH antigens [4]. CareStart™RDT has been evaluated previously in field settings using PCR-corrected microscopy as reference, showing better sensitivity for microscopy [16]. In a few studies the microscopy standard was not corrected by PCR [17]. High sensitivity (94%) were noted when parasitaemia were above 100 parasites/μL. The sensitivity increased with the increasing parasitaemia, reaching 99% when parasitaemia was up to 1000 parasites/μL, while sensitivity was only 88% when parasitaemia was fewer than 100 parasites/μL [4]. In the study described here, there were 8 samples with parasitaemia under 100 parasites/μL, and 23 samples had parasitaemia fewer than 1,000 parasites/μL. Since all positive microscopy were also positive in CareStart™RDT and confirmed by PCR, the sensitivity against microscopy was 100% irrespective to range of parasitaemia. However, at the PCR level, sensitivity of CareStart™RDT was significantly higher. A previous study reported superior sensitivity of RDT over microscopy (including expert microscopy) [5]. In a field study performed in unstable malaria transmission, performance of CareStart™RDT has shown a sensitivity of 85.6% and specificity of 92.5% when compared to gold standard microscopy; the sensitivity increased with increasing parasite densities, achieving 95.8% when parasite density was higher than 5000 parasites/μL [18, 19]. However, in another study, CareStart™RDT showed better sensitivity (99.4%) and specificity (96.0%) while it did not show change in sensitivity with decreasing parasitaemia [20]. Unlike when compared to microscopy, RDTs showed lower sensitivity against PCR in a holo-endemic area where malaria transmission occurs throughout the year [19].

Despite CareStart™RDT detecting more true positive samples than microscopy in this study, there were also some false positive records. These false positives may be due to the persistence of the HRP2 antigen after treatment [21]. However, it has been shown that CareStart™RDT false positivity decreases quickly after successful treatment [20]. Moreover, it has been suggested that rheumatoid factor can produce false positive due to binding IgG [22]. Although the use of IgM is supposed to reduce the issue of rheumatoid factor cross-reactions [15], it is not known which type of antibody was used to coat CareStart™RDT.

Many other factors can affect the performance of RDTs. False-negative can be observed when parasites fail to express the target antigen (gene deletion) or when the parasite express a variant of the protein (polymorphism) which can affect the antigen–antibody binding. *PfHRP2* gene sequence of parasites varies depending on the geographic locations [15]. In Senegal, until recently, *PfHRP2* polymorphism did not affect the performance of HRP2-based RDTs [23] and no *PfHRP2* gene deletion was observed. However, in Mali, a neighbouring country of Senegal, parasites lacking *PfHRP2* have been reported to cause false-negative results [24] suggesting a need for a larger investigation.

Unlike PfHRP2, the pLDH protein does not persist in the blood after effective treatment with anti-malarial [20]. Thus, the protein has been proposed as a target for monitoring parasite responses to treatment and for predicting treatment failure [15, 20]. Moreover, the pLDH protein seems not to be subject of antigenic variation [15]. Taken together, pLDH-based RDTs should be an alternative approach in case of *PfHRP2* gene deletion.

However, pLDH-based RDTs have several limitations. pLDH-based tests have decreased sensitivity at low parasitaemia [7]. The positivity rate of pLDH in this study was very low even at relatively high parasitaemia when compared to other studies [4]. The storage and transportation conditions could affect the performance of RDTs in field conditions. In fact, the quality of RDTs may show poor performances when they are exposed to heat and humidity [6]. pLDH is especially sensitive to those tropical conditions than HRP2 [7]. Therefore, quality control of procured RDTs is essential to minimize false negative pLDH results.

## Conclusions

Although RDT usefulness in low transmission areas or in the detection of low parasite density infections is being questioned, CareStart™RDT showed good sensitivity comparable to that performed by expert microscopist. However, in the wake of reported HRP2 deletion in other countries, microscopy should always accompany the use of RDT. Combination of RDT and microscopy together with the evaluation of malaria RDTs over time should be a powerful tool for diagnosing malaria in endemic countries.

## Abbreviations

CT: threshold cycle
DNA: deoxyribonucleic acid
EDTA: ethylenediaminetetraacetic acid
ECA: external competency assessment
Ig: immunoglobulin
LDH: lactate dehydrogenase
NMCP: National Malaria Control Programme
NPV: negative predictive value
PET-PCR: photo-induced electron transfer-polymerase chain reaction
PfHRP: *Plasmodium falciparum* histidine-rich protein
pLDH: *Plasmodium* lactate dehydrogenase
PPV: positive predictive value
RDT: rapid diagnostic test
WHO: World Health Organization
